# Occupational Risk of Low-Level Blast Exposure and TBI-Related Medical Diagnoses: A Population-Based Epidemiological Investigation (2005–2015)

**DOI:** 10.3390/ijerph182412925

**Published:** 2021-12-08

**Authors:** Jennifer N. Belding, Robyn Englert, James Bonkowski, Cynthia J. Thomsen

**Affiliations:** 1 Leidos, Inc., San Diego, CA 92106, USA; robyn.m.englert.ctr@mail.mil (R.E.); james.f.bonkowski3.ctr@mail.mil (J.B.); 2 Naval Health Research Center, San Diego, CA 92106, USA; cynthia.j.thomsen.civ@mail.mil

**Keywords:** blast, low-level blast, high-level blast, TBI, concussion, overpressure, military, epidemiology, healthcare

## Abstract

Because traumatic brain injury (TBI)—most often caused by exposure to high-level blast (HLB)—is a leading cause of medical evacuations of deployed U.S. service members in recent conflicts, researchers seek to identify risk factors for TBI. Previous research using self-reported data has identified low-level blast (LLB) as one such risk factor and suggests an association with susceptibility to and symptoms associated with TBI. This article presents a population-based study of all branches of military service that examines the association between occupational risk for LLB and both clinically diagnosed TBIs—from concussions to severe and penetrating TBIs—and conditions commonly comorbid with concussion. Using archival medical and career records from >2 million service members between 2005–2015, this work demonstrates that occupational risk of LLB is associated with any TBI, mild TBI, moderate TBI, cognitive problems, communication problems, hearing problems, headaches, any behavioral health condition, anxiety, drug abuse/dependence, alcohol abuse/dependence, delirium/dementia, posttraumatic stress disorder, post-concussive syndrome, tinnitus, fatigue, and migraines. Understanding the full scope of the effects of LLB on service members will help ensure the health and readiness of service members and may influence both military policy and clinical practice guidelines for blast-induced injuries.

## 1. Introduction

More than 50,000 members of the U.S. Armed Forces were wounded while deployed in support of Operations Enduring Freedom, Iraqi Freedom, and New Dawn [1], and many of their injuries resulted from being in close proximity to detonated explosive devices [2,3,4]. When detonated, these explosives subject service members to a shockwave and corresponding increase in ambient pressure (called overpressure) [5]. Exposure to such explosions is the leading cause of deployment-related traumatic brain injuries (TBIs) [4,6], which are themselves one of the leading causes of deployment-related medical evacuations [7]. Exposure to high-level blast (HLB) can also result in other health conditions (e.g., traumatic amputations, posttraumatic stress disorder [PTSD]) and is thus a significant threat to service member health, well-being, and readiness [6,8]. As a result, a great deal of research seeks to identify the risk factors associated with TBI and other blast-induced injuries [9,10,11].

In addition to research on the effects of exposure to HLB on TBI, there is a growing body of scientific research on low-level blast (LLB) [12,13,14]. LLB is similar to HLB in that both are forms of overpressure exposure generated by the detonation of munitions. The term HLB is used to denote overpressure that results from incoming munitions such as improvised explosive devices, whereas LLB denotes overpressure that results from outgoing munitions, such as that generated by firing certain weapons systems (e.g., the Carl Gustav) [5]. Although there is a strong body of literature identifying the long-term consequences of HLB, research on the adverse outcomes associated with LLB is still in its infancy. This research suggests that exposure to LLB has the potential to harm service members [11,12,13,14]. The limited research on LLB began in the 1980s and 1990s with investigations assessing risk for pulmonary injury, while more recent research has examined predominantly neurological outcomes [13]. According to findings from a scoping review of research articles published between 2000 and 2019, recent studies suggest that LLB exposure is associated with subclinical symptoms associated with concussion, tinnitus, and hearing-related concerns, as well as a variety of indicators of potential brain damage [13].

However, previous research on LLB is limited in several ways. Studies on human participants have relied on very small samples (typically fewer than 40 participants in military and law enforcement samples) [15,16,17,18,19,20,21,22]. Additionally, these studies often investigated self-reported subclinical symptoms, which may not correspond to clinically diagnosable injuries [23]. Studies have also been limited in their ability to investigate conditions that are associated or commonly comorbid with TBI (e.g., PTSD) [24]. Thus, there is a pressing need to conduct large-scale epidemiological investigations of the effects of LLB exposure on service member health, well-being, and readiness.

In an attempt to address this need, two research teams have conducted epidemiological investigations of the sequelae associated with occupational risk of LLB [25,26,27,28]. During their military service, many service members are required to be in close proximity to the firing of certain weapons systems that are known to generate significant amounts of LLB, but the extent of this exposure varies widely, depending on the unique duties of each military occupational specialty (e.g., artillery, personnel and administration). Based on this variation in exposure, these research teams have suggested that service members can be classified into groups with relatively high, moderate, or low risk of exposure to LLB. These classifications can then be used as proxies to examine differences in medical and career outcomes as a function of LLB exposure. For example, researchers leveraged data active duty enlisted Marines had reported on the Post-Deployment Health Assessment (PDHA) and Post-Deployment Health Reassessment (PDHRA), two mandatory surveys that must be completed at return from deployment and approximately 6 months later, respectively. The data demonstrated that Marine Corps personnel working in occupations at high risk for LLB were significantly more likely than those in low-risk occupations to sustain a mild TBI (also called concussion) following exposure to a potential TBI-inducing event [26]. Furthermore, the data showed that those working in high-risk occupations were significantly more likely to report neurological symptoms for which they sought care during deployment following concussion [25] and that these symptoms often persisted approximately 6 months later [27]. However, this work was limited only to Marines who completed the PDHA, and it relied solely on self-report measures that may be subject to recall and response biases.

Building on this prior work, Carr et al. [28] sought to address these limitations by conducting an epidemiological investigation using medical and career records, including clinical diagnoses of injury. Using a sample of approximately 100,000 U.S. Army soldiers and a matched cohort design, they found that occupational risk of LLB was associated with increased likelihood of being diagnosed with TBI and tinnitus. Notably, they showed that these associations were significantly stronger for those with more time in service. However, this work was limited in that it involved only members of the U.S. Army and did not distinguish TBIs by severity, which ranges from mild to moderate, severe, and penetrating, based on the duration of the loss or alteration of consciousness and mechanism of injury [29]. Furthermore, although mild TBIs are far more common than their more severe counterparts [30], Carr and colleagues did not examine specific conditions commonly comorbid with concussion.

Despite great strides in attempting to understand the consequences associated with occupational exposure to LLB, there is a need for a population-based study including all branches of U.S. military service that examines the association between occupational risk of LLB and clinically diagnosed TBIs. In particular, there is a need for a study that can differentiate between mild, moderate, severe, and penetrating TBIs and can simultaneously examine conditions commonly comorbid with concussion. The present research sought to fill this gap by examining whether occupational risk of LLB is associated with greater risk of clinically diagnosable TBI—from concussions to severe and penetrating TBIs—and conditions commonly comorbid with concussion. This research also sought to examine whether such associations may be more pronounced among those with greater time in service.

It was hypothesized that occupational risk would be associated with greater risk of any TBI, but specifically TBIs of lower severity (e.g., mild TBI, moderate TBI), as well as behavioral health conditions, including PTSD and depression. Drawing on findings discussed in the scoping review [13], it was also hypothesized that occupational risk of LLB would be associated with greater risk of post-concussive syndrome, tinnitus, fatigue, and migraines. Consistent with prior research, it was theorized that the concept of injury ranges on a continuum from non-injured to severely injured, and that exposure to LLB is considered incremental and cumulative in nature and may push people along this continuum thereby increasing their risk of injury [5]. Therefore, it was predicted that associations between exposure to LLB and clinical diagnoses would be more likely to emerge with greater time in service and should be largely absent among service members at baseline (i.e., with less than 1 year of service).

## 2. Materials and Methods

### 2.1. Data Source

Data were accessed and pulled using the Naval Health Research Center (NHRC) Career History Archival Medical and Personnel System (CHAMPS). This database maintains a longitudinal record of service members’ pay-affecting career records (including accession into military service, duty station changes, military occupation, pay grade, and discharge) and medical data (including dates and diagnoses using the International Classification of Diseases, 9th Revision or procedure codes) for medical encounters at both military treatment facilities and purchased care (i.e., civilian) settings [31]. Due to a combination of Health Insurance Portability and Accountability Act (HIPAA) regulations and limitations based on the CHAMPS data sharing agreement, data used for this study cannot be shared with personnel outside of NHRC, though study materials and code are available from the authors upon request. This study was approved by the NHRC Institutional Review Board (protocol no. NHRC.2016.0024) and a waiver of informed consent was granted.

### 2.2. Participants

**Figure 1:**
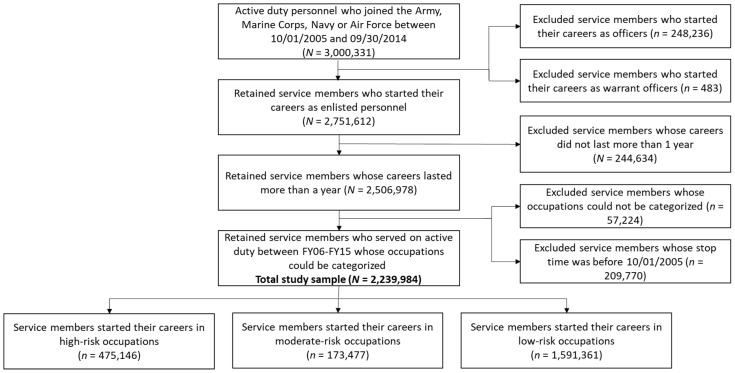
Flow diagram.

Data were obtained for active-duty service members who initially joined the U.S. Army, Navy, Marine Corps, or Air Force between 1 October 2005 (fiscal year 2006) and 30 September 2014 (fiscal year 2014); records for enlisted personnel who served on active duty for at least 1 year were retained. Participants whose occupations could not be classified were excluded (see Coding Military Occupations section below and Figure 1). Because the participant population was limited to new accessions during this 10-year period, the maximum amount of time in service for these participants was 10 years.

### 2.3. Stop Rules

Because changes in an individual’s branch of service (e.g., Marine Corps to Navy), pay grade (e.g., enlisted to officer), or military occupational specialty risk category (e.g., high to low) could also alter how each participant was categorized during analysis, medical and career data for service members was followed until the earliest of the following events occurred: (1) they changed from one branch of service to another; (2) they converted from an enlisted pay grade to a warrant officer or officer pay grade; (3) their occupation changed, resulting in a risk categorization shift (as subsequently described); (4) they were discharged from military service for at least 30 days; or (5) the study end date of 31 December 2015. Data for participants after any of these events occurred were censored and thus not included in subsequent analysis.

### 2.4. Coding Military Occupations

Because there were more than 11,000 official service-specific job titles in use across the four branches of service during the study time period, occupations were identified at the broader Department of Defense (DoD) level, which includes 187 categories. Using these, participants were grouped into occupations with relatively low, moderate, or high risk of LLB exposure, consistent with previous epidemiological research [25,26,27]. High-risk occupations included general armor and amphibious; artillery and gunnery; aviation ordnance; general combat engineering; general combat operations control; explosive ordnance disposal/Underwater Demolition Team; expeditionary medical services; general infantry; infantry, gun crews, seamanship specialists; military training instructor; missile artillery operating crew; rocket artillery; and special forces. Moderate-risk jobs included ammunition repair, artillery repair, counterintelligence, general armament maintenance, general law enforcement, independent duty hospital services, operational intelligence, security guards, and tracked vehicles. The remaining 160 occupations were categorized as low risk.

Unlike previous research, which was based on self-reported occupation, the present research used official military occupations (i.e., DoD occupational codes) recorded at least 12 months following initial accession to military service. Because service members typically complete regular basic training that is agnostic to their ultimate military occupation during their first 12 months of service, classification began at 12 months after accession. When occupational codes specified a rank rather than a specific occupation (e.g., “seaman”), the subsequent occupation was used. Because service members can switch occupations over time, complicating interpretation of whether medical and career sequelae are due to occupational differences, data for those whose changes in occupation would result in a different risk categorization were censored. Data for those who switched occupations but remained in the same risk categorization level (e.g., infantry [high risk] to artillery [high risk]) were retained. Total time in occupation was determined using the number of days, converted to years, between their first occupation on record and their final applicable event as noted in the stop rules above.

### 2.5. Medical Diagnoses

Diagnoses of TBI were identified in accordance with case surveillance criteria established by the Armed Forces Health Surveillance Branch (AFHSB). Diagnoses of any TBI, mild TBI (which includes post-concussive syndrome per AFHSB criteria), moderate TBI, severe TBI, penetrating TBI, and unclassified TBI were retained. It was possible for each service member to have been diagnosed with more than one type of TBI during the study period. Furthermore, given the direct focus on concussion in the present research, additional relevant conditions were recorded using case criteria established in a recent RAND report on concussion [32]. Diagnoses commonly comorbid with concussion that were identified for analysis included alteration in mental status, cognitive problems, communication disorders, dizziness/vertigo, gait and coordination problems, headache, hearing problems, non-headache pain, skin sensation disturbances, sleep disorders/symptoms, smell and taste disturbances, syncope/collapse, and vision problems. Behavioral health conditions that were identified for analysis included adjustment disorders, anxiety disorders, acute stress disorders, alcohol abuse/dependence, attention-deficit/hyperactivity disorder (ADD/ADHD), bipolar disorder, delirium/dementia, depression, drug abuse/dependence, personality disorders, and PTSD. For exploratory purposes, several additional diagnoses thought to be specifically related to LLB were also retained, including specific diagnoses of post-concussive syndrome (310.2), tinnitus (388.3), fatigue (780.7), and migraines (346). For each medical diagnosis or category of interest (see Supplement), the date of the earliest diagnosis on record, as well as the earliest date recorded in inpatient and outpatient settings, respectively, were recorded.

### 2.6. Statistical Analyses

The number and proportion of service members with each condition were computed. Whether or not participants were diagnosed (in either inpatient or outpatient settings) with each condition of interest was regressed on occupational risk for LLB, time in occupation, and their interaction, while controlling for sex and branch of service using Cox proportional hazards models. When significant interactions emerged, we decomposed the interaction by stratifying by time in service using the same categories as Carr and colleagues [28]: less than 1 year in service, 1–7 years, and 7–10 years, which can roughly be interpreted as baseline, early career, and mid-career, respectively. Due to the number of comparisons and large sample size, a threshold of *p* < 0.001 was used to determine statistical significance. The 95% confidence interval for each adjusted hazard ratio is presented.

## 3. Results

### 3.1. Frequencies

Frequencies for each of the medical diagnoses of interest are shown in Table 1. Approximately 7.5% of the sample had a diagnosis of TBI; approximately 6.8% had a mild TBI. Of the conditions commonly comorbid with TBI, more than half of the sample (59.3%) had been diagnosed with non-headache pain, and 18.6% had been diagnosed with a sleep disorder or symptom. Diagnoses of smell and taste disturbances were reported too infrequently to be released due to HIPAA regulations and thus were excluded from subsequent analyses. Approximately 28.5% of the sample had at least one diagnosed behavioral health condition; the most frequently diagnosed were adjustment disorders, anxiety disorders, and alcohol abuse/dependence. Additionally, 1.3% of the sample had been diagnosed with post-concussive syndrome, 3.7% with tinnitus, 5.8% with fatigue, and 5.6% with migraines.

### 3.2. TBI Diagnoses

**Figure 2:**
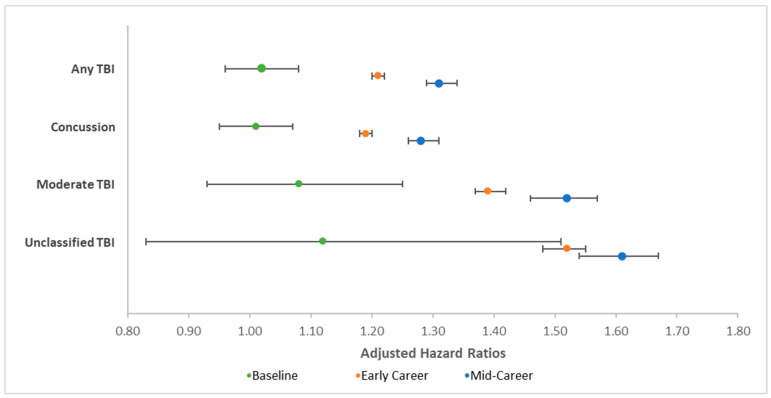
Graph of adjusted hazard ratios depicting decomposition of significant interaction between occupational risk of low-level blast (LLB) and time in risk for traumatic brain injuries (TBIs) of different severity. These adjusted hazard ratios represent the odds of being diagnosed with the conditions denoted as a function of occupational risk of LLB. Error bars represent 95% confidence intervals.

Occupational risk and time in service were independently associated with each of the TBI diagnoses of interest (see Table 2). Specifically, those in high-risk occupations were significantly more likely to be diagnosed with each of the TBI diagnoses than were their lower risk counterparts (adjusted hazard ratios range = 1.26–1.58). Additionally, years at risk was significantly associated with lower risk of TBI diagnoses (adjusted hazard ratios = 0.71–0.92). The interaction of repetitive LLB exposure and years at risk was significant for any TBI, mild TBI, moderate TBI, and unclassified TBI, but not for severe or penetrating TBI. When significant interactions were decomposed as a function of time in risk (see Table 3), there were no significant differences in any TBI, mild TBI, and moderate TBI as a function of occupational risk of LLB at baseline. However, occupational risk of LLB was associated with greater risk of any TBI, mild TBI, and moderate TBI among early career service members, which was further elevated among mid-career service members (see Figure 2).

### 3.3. Commonly Comorbid Diagnoses

Of the 12 conditions that are commonly comorbid with TBI, occupational risk of LLB was associated with greater risk of altered mental status, cognitive problems, communication disorders, headaches, and hearing problems (see Table 2). Occupational risk of LLB was associated with significantly reduced risk of dizziness/vertigo, non-headache pain, sleep disorders, and vision problems. Occupational risk was not independently associated with gait and coordination problems, skin sensation disturbances, or syncope and collapse. Time in occupational risk was associated with significantly reduced risk of all the commonly comorbid conditions. The interactions of occupational risk of LLB and time in risk were significant for six of the thirteen commonly comorbid conditions, including cognitive problems, communication disorders, headache, hearing problems, non-headache pain, and sleep disorders and symptoms.

**Figure 3:**
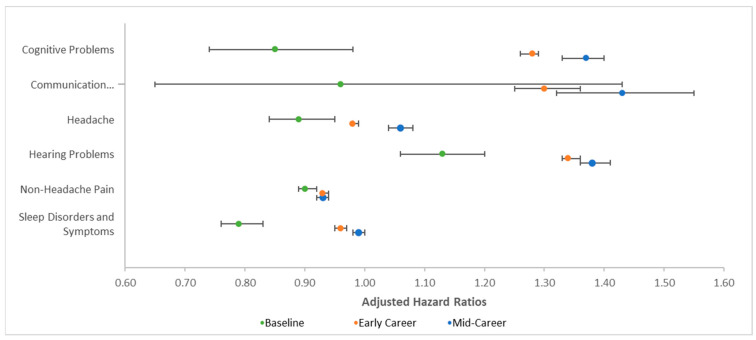
Graph of adjusted hazard ratios depicting decomposition of significant interaction between occupational risk of low-level blast (LLB) and time in risk for conditions commonly comorbid with mild traumatic brain injury. Adjusted hazard ratios represent the odds of being diagnosed with the conditions denoted as a function of occupational risk of LLB. Error bars represent 95% confidence intervals; all error bars are included, but some are not visually apparent because they are so close to the point estimate (e.g., lower bound for headaches).

Decomposition of these interactions is presented in Table 3 and Figure 3. Although occupational risk was not associated with cognitive problems at baseline, occupational risk was associated with significantly greater risk of cognitive problems among early career and mid-career service members, with a significant difference between early and mid-career. Similarly, occupational risk was not associated with communication disorders at baseline, but it was associated with greater risk at early and mid-career (which did not significantly differ). Occupational risk was associated with significantly lower risk of headaches among those at baseline and early career, but this pattern reversed over time and occupational risk was associated with significantly greater risk of headaches among those in mid-career. Unsurprisingly, occupational risk was significantly associated with hearing problems at all three time points and grew progressively stronger over time. Interestingly, occupational risk was associated with significantly lower risk of non-headache pain at all three time points, but this effect was strongest at baseline. Additionally, occupational risk was associated with significantly lower risk of sleep disorders and symptoms at baseline and early career, but it was not significantly associated at mid-career.

### 3.4. Behavioral Health Diagnoses

Occupational risk of LLB was associated with significantly greater risk of any behavioral health condition as well as anxiety disorders, alcohol abuse/dependence, delirium/dementia, and PTSD (see Table 2). Occupational risk of LLB was associated with reduced risk of ADD/ADHD, bipolar disorder, and personality disorders. There was no association of occupational risk of LLB with adjustment disorders, acute stress disorders, depression, or drug abuse/dependence. Additionally, time in occupational risk was associated with significantly reduced risk of all the behavioral health conditions. There were significant occupational risk and time in risk interactions for any behavioral health condition and eight of the eleven specific conditions (except acute stress disorders, bipolar disorder, and depression).

**Figure 4:**
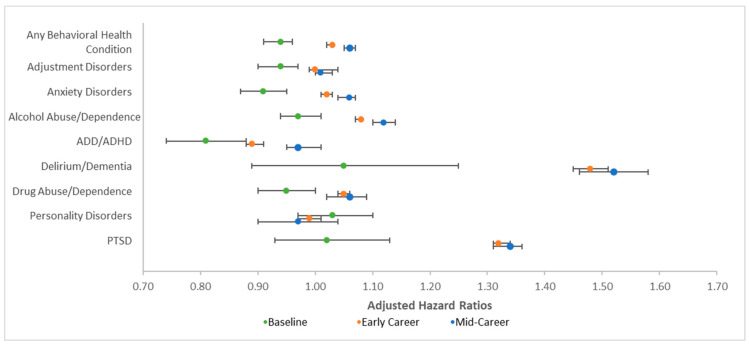
Graph of adjusted hazard ratios depicting decomposition of significant interaction between occupational risk of low-level blast (LLB) and time in service for behavioral health conditions. Adjusted hazard ratios represent the odds of being diagnosed with the conditions denoted as a function of occupational risk of LLB. Error bars represent 95% confidence intervals; all error bars are included, but some are not visually apparent as they are so close to the point estimate (e.g., upper bound for any behavioral health condition). ADD/ADHD, attention-deficit disorder/attention-deficit/hyperactivity disorder; PTSD, posttraumatic stress disorder.

Decomposition of these interactions is presented in Table 3 and Figure 4. Whereas occupational risk was associated with lower risk of any behavioral condition, anxiety disorders, and drug abuse/dependence at baseline, this pattern reversed, and occupational risk was associated with greater risk at early and mid-career time points. Additionally, although occupational risk was associated with lower risk of adjustment disorders and ADD/ADHD at baseline, these patterns attenuated over time. Similarly, whereas occupational risk was not associated with differences in alcohol abuse/dependence, delirium/dementia, and PTSD at baseline, it was associated with significantly greater risk of these conditions at early and mid-career time points.

### 3.5. LLB-Related Diagnoses

**Figure 5:**
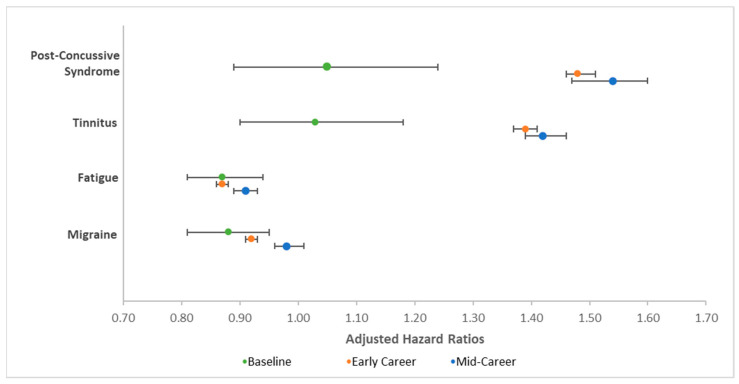
Graph of adjusted hazard ratios depicting decomposition of significant interaction between occupational risk of low-level blast (LLB) and time in risk for conditions hypothesized to be related to LLB. Adjusted hazard ratios represent the odds of being diagnosed with the conditions denoted as a function of occupational risk of LLB. Error bars represent 95% confidence intervals.

Occupational risk, time in risk, and their interaction were significant for each of the four exploratory diagnoses hypothesized to be related to LLB (see Table 2). While occupational risk of LLB was associated with greater risk of post-concussive syndrome and tinnitus, it was associated with less risk of migraines and fatigue. Additionally, time in service was once again associated with less risk for each of the four conditions. However, the significant interaction of occupational risk and time in service for each condition suggests further nuance to these findings (see Table 3 and Figure 5). There was no significant effect of occupational risk on post-concussive syndrome or tinnitus at baseline, but occupational risk was associated with higher risk of post-concussive syndrome and tinnitus at the early and mid-career time points. Occupational risk was associated with significantly lower risk of fatigue at all three time points, but this finding attenuated significantly over time. Similarly, whereas occupational risk was associated with significantly lower risk of migraines at both baseline and early career time points, this difference was no longer significant at the mid-career time point.

## 4. Discussion

While previous research has suggested that there is a relationship between exposure to LLB and symptoms associated with concussion, previous work has been limited by reliance on small sample sizes and self-report measures of symptoms [13]. To address these limitations, the present study used a large sample and official clinical diagnoses to investigate whether occupational risk of LLB exposure is associated with greater risk of any TBI as well as specific severities of TBI, conditions commonly comorbid with concussion (including behavioral health conditions), and specific conditions hypothesized to be related to overpressure exposure (i.e., tinnitus, post-concussive syndrome, fatigue, and migraines). To achieve this, archival medical and career records from over two million service members who served on active duty between 2005 and 2015 were analyzed for likelihood of being diagnosed with these conditions. Because we hypothesized that adverse outcomes are likely to emerge with greater cumulative exposure to occupational LLB, we examined whether there was a significant interaction between occupational risk for LLB and time spent working in that occupation.

Occupational risk of LLB exposure was associated with any, mild, moderate, severe, penetrating, and unclassified TBI. However, the key hypothesis centered around whether such effects were moderated by time in occupation, which could be indicative of a dose–response relationship between LLB and adverse health outcomes. Our findings demonstrated that occupational risk for LLB may be associated with greater susceptibility to concussion and moderate TBIs, but not severe or penetrating TBIs, which are likely caused by sufficiently forceful events or impacts to the head to result in TBI regardless of occupational exposure to LLB. Interestingly, the effect of occupational risk for LLB exerted a stronger effect on moderate TBIs compared with concussions, which may be partially explained by the fact that people often do not seek medical care for a concussion, thus such effects would not be observed in studies using healthcare reimbursement records [32].

In addition to examining the association between occupational risk of LLB and diagnoses of TBI, the present study also examined conditions commonly comorbid with concussion, including behavioral health conditions. Findings demonstrated that more time spent in occupations at high risk of LLB was associated with cognitive problems, communication disorders, headaches, hearing problems, non-headache pain, and sleep disorders and symptoms. Furthermore, longer time spent at high occupational risk of LLB was associated with greater risk of being diagnosed with any behavioral health condition, anxiety, drug abuse/dependence, alcohol abuse/dependence, delirium/dementia, and PTSD.

Additionally, to expand on previous findings from studies involving breaching instructors and law enforcement personnel, we examined several conditions that were hypothesized to be related to LLB [11,12,13,14]. One of the most consistent findings to date regarding the association between LLB and adverse outcomes involves tinnitus [14,24,28,33]. While occupational risk was not associated with differences in tinnitus at baseline, it was associated with a 39% greater risk of tinnitus among those early in their military careers (i.e., with 1–7 years of service) and 42% among those in their mid-career (those with 7–10 years of service), though the latter two were not significantly different from each other. Stated differently, those working for 7–10 years in occupations marked by LLB were not significantly worse off than those with 1–7 years in service with respect to tinnitus diagnoses. This may be partially explained by DoD policy requiring annual auditory exams using objective, validated auditory screenings that can be used to diagnose tinnitus and other hearing-related conditions.

Consistent with our previous work using PDHA and PDHRA data [25,26,27], these analyses suggest that occupational risk of LLB exposure was associated with a 57% increase in risk of being diagnosed with post-concussive syndrome. However, this effect was moderated by time, indicating that while occupational risk was unrelated to the likelihood of being diagnosed with post-concussive syndrome at baseline, longer time in high-risk occupations was associated with greater risk of post-concussive syndrome.

Findings regarding fatigue and migraines, two other conditions that have been reported to be associated with LLB [5,12,14], were also interesting. They suggest that cumulative exposure to repetitive LLB may be associated with fatigue, although those working in high-risk occupations were less likely to be diagnosed with these conditions overall; these findings parallel the effects demonstrated for sleep disorders and symptoms. Similarly, although occupational risk was associated with lower likelihood of being diagnosed with migraines at baseline and in early career, this difference was no longer significant among those in mid-career; this can be interpreted as suggesting that occupational risk of LLB may indeed be associated with greater risk of migraines over time.

Taken together, these findings add to a growing body of evidence which suggests that occupational exposure to LLB is associated with adverse health outcomes [13]. Specifically, this work suggests that occupational risk of LLB may be associated with any TBI, mild TBI, moderate TBI, cognitive problems, communication problems, hearing problems, headaches, any behavioral health condition, anxiety, drug abuse/dependence, alcohol abuse/dependence, delirium/dementia, PTSD, post-concussive syndrome, tinnitus, fatigue, and migraines. Although it is possible to recover from many of these conditions, service members facing these issues may also have additional ramifications for their well-being, including potentially affecting their job performance, quality of life, and more. While it is beyond the scope of the present investigation, the research team is currently conducting another study examining the effects of occupational risk of LLB on performance and career outcomes, which will be able to shed further light on such potential consequences of LLB exposure.

The only other published work to date that has examined the association between occupational risk for LLB and official clinical diagnoses is work by Carr and colleagues, which demonstrated that occupational exposure to LLB is associated with TBI in general, post-concussive syndrome, and tinnitus [28]. However, that previous work was limited in that it examined only Army service members and included only a broad TBI category that did not follow standard AFHSB case surveillance definitions or stratify by TBI severity. The study presented here expands on previous work by including members from all branches of service, and by conducting a thorough examination of TBIs of different severities, as well as physical and behavioral health conditions commonly comorbid with concussion.

Despite the strengths of this approach, several limitations should be considered. First, this study included only those who newly joined the military between 2005 and 2015 and thus followed service members for a maximum period of 10 years. It is possible that the adverse health effects of LLB may have a longer latency period, and an understanding of the full scope of such effects would require additional follow-up time. Second, although the present work used official clinical diagnoses, even these data may be limited because they assume that service members sought medical care that was paid for by TRICARE. Unfortunately, these data are not able to account for TBIs or associated conditions that were sustained prior to military service and do not include diagnoses recorded in deployed environments. Furthermore, the use of military occupations as a proxy for repetitive exposure to LLB is not precise and may confound LLB with other factors (e.g., operational tempo) that could contribute to health outcomes, a point that has received sufficient articulation elsewhere [25,26,27].

Because exposure to LLB is an inherent occupational risk associated with certain military occupations, additional research is still needed to understand the scope of the effects of such exposure on warfighter health and well-being. For example, additional research could examine whether the diagnoses examined in the current research may also be associated with adverse career outcomes, including medical or administrative separation from military service. Additionally, a longer time frame that would follow service members throughout their full military careers—and possibly beyond separation from service—would enhance our understanding of the long-term outcomes associated with LLB, including end-of-life conditions such as dementia. Furthermore, the field would benefit from additional research that examines subclinical symptoms and clinical diagnoses simultaneously, particularly using prospective longitudinal designs.

## 5. Conclusions

This work provides additional evidence that occupational risk of LLB exposure may be associated with greater risk of clinical diagnoses, including, but not limited to, concussion, moderate TBI, and post-concussive syndrome. In particular, this work is the first to demonstrate the association between occupational risk of LLB and clinical diagnoses of injury across all branches of U.S. military service. It is also the first to examine severity of TBI and conditions commonly comorbid with concussion. Understanding the full scope of the effects of LLB on service member brain health and overall well-being is important to ensure the health and readiness of members of the U.S. Armed Forces. Despite strides in development of personal protective equipment and other mitigation strategies (e.g., stand-off distances), it should be assumed that warfighters will continue to be exposed to weapons systems known to generate high levels of LLB in both training and operational environments. Through increased understanding of the full scope of the outcomes associated with such exposures, we can update safety and training protocols and clinical practice guidelines for the prevention, identification, and treatment of TBIs and related health outcomes to ensure that warfighters and veterans receive the medical care and support they need.

## Figures and Tables

**Table 1:** Frequencies of each condition across the entire sample, stratified by branch of service.

	Army		Air Force		Navy		Marine Corps		Total	
	*N*	%	*N*	%	*N*	%	*N*	%	*N*	%
**TBI**										
Any TBI	54,170	9.8	13,149	5.8	12,534	4.3	19,783	7.6	99,636	7.5
Mild TBI	48,619	8.8	12,566	5.5	11,939	4.0	18,149	6.9	91,273	6.8
Moderate TBI	10,931	2.0	1631	0.7	1705	0.6	4232	1.6	18,499	1.4
Severe TBI	326	0.1	78	<0.1	64	<0.1	149	0.1	617	<0.1
Penetrating TBI	480	0.1	111	<0.1	96	<0.1	220	0.1	907	0.1
Unclassified TBI	8834	1.6	289	0.1	317	0.1	2476	0.9	11,916	0.9
**Commonly comorbid with TBI**										
Altered mental status	10,191	1.8	2834	1.2	3327	1.1	4377	1.7	20,729	1.6
Cognitive problems	28,345	5.1	4701	2.1	2978	1.0	6084	2.3	42,108	3.1
Communication disorders	1998	0.4	488	0.2	391	0.1	855	0.3	3732	0.3
Dizziness/vertigo	3881	0.7	2250	1.0	1659	0.6	977	0.4	8767	0.7
Gait and coordination problems	372	0.1	132	0.1	132	<.01	176	0.1	812	0.1
Headache	66,531	12.0	22,686	10.0	19,120	6.5	14,319	5.5	122,656	9.2
Hearing problems	46,011	8.3	10,724	4.7	13,070	4.4	18,057	6.9	87,862	6.6
Non-headache pain	394,599	71.3	143,063	63.1	121,243	41.1	133,960	51.3	792,865	59.3
Skin sensation disturbances	633	0.1	164	0.1	141	<0.1	191	0.1	1129	0.1
Sleep disorders and symptoms	147,074	26.6	46,695	20.6	27,769	9.4	26,737	10.2	248,275	18.6
Syncope and collapse	1824	0.3	530	0.2	510	0.2	431	0.2	3295	0.2
Vision problems	22,239	4.0	11,213	4.9	9456	3.2	6622	2.5	49,530	3.7
**Behavioral health conditions**										
Any behavioral health condition	209,271	37.8	55,277	24.4	59,623	20.2	56,790	21.7	380,961	28.5
Adjustment disorders	100,531	18.2	23,061	10.2	21,077	7.1	17,789	6.8	162,458	12.2
Anxiety disorders	88,185	15.9	25,077	11.1	21,294	7.2	20,063	7.7	154,619	11.6
Acute stress disorders	495	0.1	98	<0.1	83	<0.1	104	<0.1	780	0.1
Alcohol abuse/dependence	76,904	13.9	14,758	6.5	24,919	8.5	26,119	10.0	142,700	10.7
ADD/ADHD	22,521	4.1	6349	2.8	5145	1.7	4370	1.7	38,385	2.9
Bipolar disorder	8212	1.5	1577	0.7	1887	0.6	1675	0.6	13,351	1.0
Delirium/dementia	11,058	2.0	1619	0.7	1380	0.5	3607	1.4	17,664	1.3
Depression	41,159	7.4	11,343	5.0	12,057	4.1	10,403	4.0	74,962	5.6
Drug abuse/dependence	36,367	6.6	2861	1.3	5535	1.9	6398	2.4	51,161	3.8
Personality disorders	8631	1.6	2427	1.1	3721	1.3	3112	1.2	17,891	1.3
PTSD	40,711	7.4	5056	2.2	4510	1.5	10,065	3.9	60,342	4.5
**Blast-associated conditions**										
Post-concussive syndrome	10,722	1.9	1560	0.7	1309	0.4	3501	1.3	17,092	1.3
Tinnitus	28,007	5.1	7282	3.2	4407	1.5	9818	3.8	49,514	3.7
Fatigue	36,579	6.6	21,931	9.7	11,730	4.0	7548	2.9	77,788	5.8
Migraine	38,378	6.9	15,215	6.7	13,256	4.5	7978	3.1	74,827	5.6

ADD/ADHD, attention-deficit disorder/attention-deficit/hyperactivity disorder; PTSD, posttraumatic stress disorder; TBI, traumatic brain injury.

**Table 2:** Results of survival analyses, controlling for sex and branch of service.

	Occupational Risk				Years at Risk				Interaction			
	HR	LL	UL	*p*	HR	LL	UL	*p*	HR	LL	UL	*p*
**TBI**												
Any TBI	1.29	1.28	1.30	*	0.91	0.91	0.91	*	1.03	1.03	1.03	*
Mild TBI	1.26	1.25	1.27	*	0.92	0.91	0.92	*	1.03	1.02	1.03	*
Moderate TBI	1.49	1.46	1.52	*	0.85	0.85	0.86	*	1.04	1.03	1.05	*
Severe TBI	1.39	1.20	1.60	*	0.71	0.67	0.75	*	1.02	0.97	1.06	0.46
Penetrating TBI	1.44	1.30	1.60	*	0.81	0.77	0.84	*	1.00	0.96	1.03	0.88
Unclassified TBI	1.58	1.55	1.62	*	0.76	0.75	0.77	*	1.03	1.02	1.04	*
**Commonly comorbid with TBI**												
Altered mental status	1.08	1.05	1.10	*	0.77	0.76	0.78	*	1.01	1.00	1.01	0.17
Cognitive problems	1.35	1.33	1.37	*	0.77	0.76	0.77	*	1.04	1.03	1.04	*
Communication disorders	1.41	1.35	1.47	*	0.78	0.76	0.80	*	1.05	1.03	1.06	*
Dizziness/vertigo	0.94	0.91	0.97	0.001	0.92	0.90	0.93	*	0.99	0.98	1.00	0.22
Gait and coordination problems	1.12	1.00	1.25	0.05	0.85	0.81	0.89	*	1.00	0.96	1.03	>0.99
Headache	1.03	1.02	1.04	*	0.87	0.87	0.88	*	1.02	1.02	1.03	*
Hearing problems	1.38	1.37	1.39	*	0.89	0.88	0.90	*	1.02	1.01	1.02	*
Non-headache pain	0.92	0.92	0.92	*	0.94	0.94	0.94	*	1.00	0.99	1.00	*
Skin sensation disturbances	1.05	0.96	1.16	0.29	0.83	0.79	0.86	*	0.98	0.95	1.01	0.17
Sleep disorders and symptoms	0.98	0.97	0.98	*	0.82	0.81	0.82	*	1.01	1.01	1.02	*
Syncope and collapse	0.94	0.88	1.00	0.06	0.86	0.84	0.88	*	0.98	0.96	1.00	0.02
Vision problems	0.88	0.87	0.90	*	0.96	0.96	0.97	*	1.00	0.99	1.00	0.05
**Behavioral health conditions**												
Any behavioral health condition	1.03	1.03	1.04	*	0.80	0.79	0.80	*	1.01	1.01	1.01	*
Adjustment disorders	1.01	1.00	1.02	0.21	0.75	0.74	0.75	*	1.01	1.01	1.02	*
Anxiety disorders	1.05	1.04	1.06	*	0.77	0.77	0.77	*	1.02	1.02	1.02	*
Acute stress disorders	1.04	0.90	1.20	0.59	0.74	0.70	0.77	*	0.98	0.94	1.02	0.24
Alcohol abuse/dependence	1.08	1.07	1.09	*	0.79	0.79	0.79	*	1.01	1.01	1.01	*
ADD/ADHD	0.94	0.92	0.95	*	0.80	0.80	0.81	*	1.02	1.02	1.03	*
Bipolar disorder	0.94	0.91	0.98	0.001	0.61	0.60	0.62	*	1.00	0.99	1.01	0.63
Delirium/dementia	1.56	1.53	1.60	*	0.90	0.89	0.91	*	1.03	1.03	1.04	*
Depression	0.99	0.98	1.00	0.17	0.68	0.68	0.69	*	1.00	1.00	1.01	0.05
Drug abuse/dependence	0.98	0.96	1.00	0.03	0.55	0.54	0.55	*	0.99	0.99	1.00	*
Personality disorders	0.85	0.82	0.89	*	0.53	0.52	0.54	*	0.97	0.96	0.98	*
PTSD	1.37	1.36	1.39	*	0.74	0.73	0.74	*	1.03	1.03	1.04	*
**Blast-associated conditions**												
Post-concussive syndrome	1.57	1.54	1.60	*	0.91	0.90	0.91	*	1.03	1.03	1.04	*
Tinnitus	1.40	1.38	1.42	*	0.79	0.79	0.80	*	1.01	1.01	1.02	*
Fatigue	0.89	0.88	0.90	*	0.89	0.89	0.89	*	1.01	1.01	1.02	*
Migraine	0.96	0.94	0.97	*	0.90	0.89	0.90	*	1.02	1.01	1.02	*

ADD/ADHD, attention-deficit disorder/attention-deficit/hyperactivity disorder; HR, hazard ratio; LL, lower limit; PTSD, posttraumatic stress disorder; TBI, traumatic brain injury; UL, upper limit. * *p* < 0.001.

**Table 3:** Decomposition of significant occupational risk by time in service interactions ^a^.

	Baseline				Early Career				Mid-Career			
	HR	LL	UL	*p*	HR	LL	UL	*p*	HR	LL	UL	*p*
**TBI**												
Any TBI	1.02	0.96	1.08	0.55	1.21	1.20	1.22	*	1.31	1.29	1.34	*
Mild TBI	1.01	0.95	1.07	0.79	1.19	1.18	1.20	*	1.28	1.26	1.31	*
Moderate TBI	1.08	0.93	1.25	0.32	1.39	1.37	1.42	*	1.52	1.46	1.57	*
Unclassified TBI	1.12	0.83	1.51	0.46	1.52	1.48	1.55	*	1.61	1.54	1.67	*
**Commonly comorbid with TBI**												
Cognitive problems	0.85	0.74	0.98	0.03	1.28	1.26	1.29	*	1.37	1.33	1.40	*
Communication disorders	0.96	0.65	1.43	0.85	1.30	1.25	1.36	*	1.43	1.32	1.55	*
Headache	0.89	0.84	0.95	*	0.98	0.98	0.99	*	1.06	1.04	1.08	*
Hearing problems	1.13	1.06	1.20	*	1.34	1.33	1.36	*	1.38	1.36	1.41	*
Non-headache pain	0.90	0.89	0.92	*	0.93	0.93	0.94	*	0.93	0.92	0.94	*
Sleep disorders and symptoms	0.79	0.76	0.83	*	0.96	0.95	0.97	*	0.99	0.98	1.00	0.12
**Behavioral health conditions**												
Any behavioral health condition	0.94	0.91	0.96	*	1.03	1.02	1.03	*	1.06	1.05	1.07	*
Adjustment disorders	0.94	0.90	0.97	*	1.00	0.99	1.04	0.40	1.01	1.00	1.03	0.09
Anxiety disorders	0.91	0.87	0.95	*	1.02	1.01	1.03	*	1.06	1.04	1.07	*
Alcohol abuse/dependence	0.97	0.94	1.01	0.12	1.08	1.07	1.08	*	1.12	1.10	1.14	*
ADD/ADHD	0.81	0.74	0.88	*	0.89	0.88	0.91	*	0.97	0.95	1.01	0.10
Delirium/dementia	1.05	0.89	1.25	0.54	1.48	1.45	1.51	*	1.52	1.46	1.58	*
Drug abuse/dependence	0.95	0.90	1.00	0.05	1.05	1.04	1.06	*	1.06	1.02	1.09	0.002
Personality disorders	1.03	0.97	1.10	0.36	0.99	0.97	1.01	0.39	0.97	0.90	1.04	0.34
PTSD	1.02	0.93	1.13	0.69	1.32	1.31	1.34	*	1.34	1.31	1.36	*
**Blast-associated conditions**												
Post-concussive syndrome	1.05	0.89	1.24	0.57	1.48	1.46	1.51	*	1.54	1.47	1.60	*
Tinnitus	1.03	0.90	1.18	0.66	1.39	1.37	1.41	*	1.42	1.39	1.46	*
Fatigue	0.87	0.81	0.94	*	0.87	0.86	0.88	*	0.91	0.89	0.93	*
Migraine	0.88	0.81	0.95	0.001	0.92	0.91	0.93	*	0.98	0.96	1.01	0.16

ADD/ADHD, attention-deficit disorder/attention-deficit/hyperactivity disorder; HR, hazard ratio; LL, lower limit; PTSD, posttraumatic stress disorder; TBI, traumatic brain injury; UL, upper limit. ^a^ Hazard ratios are reported for occupational risk at each of the three time points and are adjusted for branch of service and sex. * *p* < 0.001.

## Data Availability

Due to HIPAA regulations and limitations stipulated in the data sharing agreements that govern the CHAMPS database, data cannot be shared with individuals outside of the Naval Health Research Center. However, documentation for database creation (e.g., R syntax) can be shared upon request.

## Supplementary Materials

The following are available online at https://www.mdpi.com/article/10.3390/ijerph182412925/s1, Table S1: ICD-9 codes used to identify each category of medical diagnoses.

## Disclaimer

I am a military service member or employee of the U.S. Government. This work was prepared as part of my official duties. Title 17, U.S.C. §105 provides that copyright protection under this title is not available for any work of the U.S. Government. Title 17, U.S.C. §101 defines a U.S. Government work as work prepared by a military service member or employee of the U.S. Government as part of that person’s official duties. Report No. 21-60 was supported by the U.S. Army Medical Research and Materiel Command, under Work Unit No. N1629. The views expressed in this article are those of the authors and do not necessarily reflect the official policy or position of the Department of the Navy, Department of Defense, nor the U.S. Government. The study protocol was approved by the Naval Health Research Center Institutional Review Board in compliance with all applicable federal regulations governing the protection of human subjects. Research data were derived from an approved Naval Health Research Center Institutional Review Board protocol, Number NHRC.2016.0024.
