# Supplementary material for: Occupational Risk of Low-Level Blast Exposure and TBI-Related Medical Diagnoses: A Population-Based Epidemiological Investigation (2005–2015)
Source: Int J Environ Res Public Health. 2021 Dec 8;18(24):12925. doi: 10.3390/ijerph182412925 (PMC8700773; doi:10.3390/ijerph182412925)
Supplement: Supplementary file 1 [file ijerph-18-12925-s001.zip › ijerph-1456852-supplementary.pdf]

International Journal of
*Environmental Research and Public Health*

MDPI

**Supplement**

Table S1: ICD-9 codes used to identify each category of medical diagnoses. Where relevant, superordinate codes listed include all subordinate codes in that category (e.g., 388.3 for tinnitus includes 388.0 [tinnitus, unspecified], 388.31 [subjective tinnitus], and 388.32 [objective tinnitus]).

| Traumatic Brain Injury | |
|---|---|
| mTBI | 310.2, 850, 850.0, 850.1, 850.11, 850.5, 850.9, 959.01, V15.5_C, V15.5_7, V15.5_2, V15.52_C, V15.52_7, V15.52_2, V15.59_C, V15.59_7, V15.59_2 |
| Moderate TBI | 800.00, 800.01, 800.02, 800.03, 800.06, 800.09, 800.10, 800.11, 800.12, 800.13, 800.16, 800.19, 800.20, 800.21, 800.22, 800.23, 800.26, 800.29, 800.30, 800.31, 800.32, 800.33, 800.36, 800.39, 800.40, 800.41, 800.42, 800.43, 800.46, 800.49, 801.00, 801.01, 801.02, 801.03, 801.06, 801.09, 801.10, 801.11, 801.12, 801.13, 801.16, 801.19, 801.20, 801.21, 801.22, 801.23, 801.26, 801.29, 801.30, 801.31, 801.32, 801.33, 801.36, 801.39, 801.40, 801.41, 801.42, 801.43, 801.46, 801.49, 803.00, 803.01, 803.02, 803.03, 803.06, 803.09, 803.10, 803.11, 803.12, 803.13, 803.16, 803.19, 803.20, 803.21, 803.22, 803.23, 803.26, 803.29, 803.30, 803.31, 803.32, 803.33, 803.36, 803.39, 803.40, 803.41, 803.42, 803.43, 803.46, 803.49, 804.00, 804.01, 804.02, 804.03, 804.06, 804.09, 804.10, 804.11, 804.12, 804.13, 804.16, 804.19, 804.20, 804.21, 804.22, 804.23, 804.26, 804.29, 804.30, 804.31, 804.32, 804.33, 804.36, 804.39, 804.40, 804.41, 804.42, 804.43, 804.46, 804.49, 850.12, 850.2, 851.00, 851.01, 851.02, 851.03, 851.06, 851.09, 851.20, 851.21, 851.22, 851.23, 851.26, 851.29, 851.40, 851.41, 851.42, 851.43, 851.46, 851.49, 851.60, 851.61, 851.62, 851.63, 851.66, 851.69, 851.80, 851.81, 851.82, 851.83, 851.86, 851.89, 852.00, 852.01, 852.02, 852.03, 852.06, 852.09, 852.20, 852.21, 852.22, 852.23, 852.26, 852.29, 852.40, 852.41, 852.42, 852.43, 852.46, 852.49, 853.00, 853.01, 853.02, 853.03, 853.06, 853.09, 854.00, 854.01, 854.02, 854.03, 854.06, 854.09, V15.5_D, V15.5_8, V15.5_3, V15.52_D, V15.52_8, V15.52_3, V15.59_D, V15.59_8, V15.59_3 |
| Severe TBI | 800.0, 800.1, 800.2, 800.3, 800.4, 800.5, 800.6, 800.7, 800.8, 800.9, 801.0, 801.1, 801.2, 801.3, 801.4, 801.5, 801.6, 801.7, 801.8, 801.9, 803.0, 803.1, 803.2, 803.3, 803.4, 803.5, 803.6, 803.7, 803.8, 803.9, 804.0, 804.1, 804.2, 804.3, 804.4, 804.5, 804.6, 804.7, 804.8, 804.9, 851.0, 851.1, 851.2, 851.3, 851.4, 851.5, 851.6, 851.7, 851.8, 851.9, 852.0, 852.1, 852.2, 852.3, |

| | |
|---|---|
| | 852.4, 852.5, 853.0, 853.1, 854.0, 854.1, 907.0, 950.1, 950.2, 950.3, V15.5_1, V15.5_B, V15.5_6, V15.52_0, V15.52_B, V15.52_6, V15.52_1, V15.59_1, V15.59_B, V15.59_6 |
| Penetrating TBI | 800.50, 800.51, 800.52, 800.53, 800.54, 800.55, 800.56, 800.59, 800.60, 800.61, 800.62, 800.63, 800.64, 800.65, 800.66, 800.69, 800.70, 800.71, 800.72, 800.73, 800.74, 800.75, 800.76, 800.79, 800.80, 800.81, 800.82, 800.83, 800.84, 800.85, 800.86, 800.89, 800.90, 800.91, 800.92, 800.93, 800.94, 800.95, 800.96, 800.99, 801.50, 801.51, 801.52, 801.53, 801.54, 801.55, 801.56, 801.59, 801.60, 801.61, 801.62, 801.63, 801.64, 801.65, 801.66, 801.69, 801.70, 801.71, 801.72, 801.73, 801.74, 801.75, 801.76, 801.79, 801.80, 801.81, 801.82, 801.83, 801.84, 801.85, 801.86, 801.89, 801.90, 801.91, 801.92, 801.93, 801.94, 801.95, 801.96, 801.99, 803.50, 803.51, 803.52, 803.53, 803.54, 803.55, 803.56, 803.59, 803.60, 803.61, 803.62, 803.63, 803.64, 803.65, 803.66, 803.69, 803.70, 803.71, 803.72, 803.73, 803.74, 803.75, 803.76, 803.79, 803.80, 803.81, 803.82, 803.83, 803.84, 803.85, 803.86, 803.89, 803.90, 803.91, 803.92, 803.93, 803.94, 803.95, 803.96, 803.99, 804.50, 804.51, 804.52, 804.53, 804.54, 804.55, 804.56, 804.59, 804.60, 804.61, 804.62, 804.63, 804.64, 804.65, 804.66, 804.69, 804.70, 804.71, 804.72, 804.73, 804.74, 804.75, 804.76, 804.79, 804.80, 804.81, 804.82, 804.83, 804.84, 804.85, 804.86, 804.89, 804.90, 804.91, 804.92, 804.93, 804.94, 804.95, 804.96, 804.99, 851.10, 851.11, 851.12, 851.13, 851.14, 851.15, 851.16, 851.19, 851.30, 851.31, 851.32, 851.33, 851.34, 851.35, 851.36, 851.39, 851.50, 851.51, 851.52, 851.53, 851.54, 851.55, 851.56, 851.59, 851.70, 851.71, 851.72, 851.73, 851.74, 851.75, 851.76, 851.79, 851.90, 851.91, 851.92, 851.93, 851.94, 851.95, 851.96, 851.99, 852.10, 852.11, 852.12, 852.13, 852.14, 852.15, 852.16, 852.19, 852.30, 852.31, 852.32, 852.33, 852.34, 852.35, 852.36, 852.39, 852.50, 852.51, 852.52, 852.53, 852.54, 852.55, 852.56, 852.59, 853.10, 853.11, 853.12, 853.13, 853.14, 853.15, 853.16, 853.19, 854.10, 854.11, 854.12, 854.13, 854.14, 854.15, 854.16, 854.19, V15.5_F, V15.5_A, V15.5_5, V15.52_F, V15.52_A, V15.52_5, V15.59_F, V15.59_A, V15.59_5 |
| Unclassified TBI | 800.04, 800.05, 800.14, 800.15, 800.24, 800.25, 800.34, 800.35, 800.44, 800.45, 801.04, 801.05, 801.14, 801.15, 801.24, 801.25, 801.34, 801.35, 801.44, 801.45, 803.04, |

| | |
|---|---|
| | 803.05, 803.14, 803.15, 803.24, 803.25, 803.34, 803.35, 803.44, 803.45, 804.04, 804.05, 804.14, 804.15, 804.24, 804.25, 804.34, 804.35, 804.44, 804.45, 850.3, 850.4, 851.04, 851.05, 851.24, 851.25, 851.44, 851.45, 851.64, 851.65, 851.84, 851.85, 852.04, 852.05, 852.24, 852.25, 852.44, 852.45, 853.04, 853.05, 854.04, 854.05, V15.5_E, V15.5_9, V15.5_4, V15.52_E, V15.52_9, V15.52_4, V15.59_E, V15.59_9, V15.59_4 |
| | |
| Conditions Commonly Comorbid with mTBI | |
| Alteration in mental status | 780.02, 780.97 |
| Cognitive problems | 799.51–55, 799.59, 780.93, 331.83 |
| Communication disorders | 784.3, 784.51, 784.59–61, 784.69 |
| Dizziness/vertigo | 386.00, 386.03, 386.10–12, 386.19, 386.2 , 386.30, 386.35, 386.40, 386.42–43, 386.50, 386.53, 386.58, 386.9, 780.4 |
| Gait and coordination problems | 781.2–3 |
| Headache | 339.10–12, 339.20–22, 339.3, 339.41–44, 339.82–85, 339.89, 339.00– 03, 346.00–03, 346.10–13, 346.20–21, 346.30, 346.40, 346.51, 346.70–73, 346.80–82, 346.90–93, 784.0 |
| Hearing problems | 388.10–12, 388.2, 388.30–32, 388.40, 388.42–45, 388.5, 388.9, 389.00–06, 389.10–22, 398.8, 398.9 |
| Non-headache pain | 307.8, 307.89, 338–338.4, 355, 355.9, 356–356.9, 357.2, 357.9, 524.6, 710–733.99 |
| Skin sensation disturbances | 782.0 |
| Sleep disorders and symptoms | 307.40–49, 327.00–.02, 327.09–15, 327.19–24, 327.26–27, 327.29, 327.30–31, 327.33, 327.35–36, 327.39, 327.40–44, 327.49, 327.51, 327.53, 327.59, 327.8, 347.00–01, 347.10, 780.50–59 |
| Smell and taste disturbances | 781.1 |
| Syncope and collapse | 780.2 |
| Vision problems | 377.75, 377.9, 378.00–01, 378.05, 378.10–11, 378.15, 378.17, 378.20– 24, 378.30–35, 378.40–43, 378.45, 378.50–55, 378.60, 379.50–52, 379.54, 379.56–58, 379.8, 379.90–93 |
| | |
| Behavioral Health Conditions | |
| Adjustment disorders | 309.0, 309.1, 309.23–24, 309.28–29, 390.3, 309.4, 309.82–83, 309.89, 309.9 |
| Anxiety disorders | 293.84, 300.00–02, 300.09, 300.20–23, 300.29, 300.3, 300.89, 300.9 |
| Acute stress disorders | 308.0–4, 308.9 |

| | |
|---|---|
| Alcohol abuse/dependence | 303.00–03, 303.90–93, 305.00–03 |
| ADD/ADHD | 314.00–01, 314.8–9 |
| Bipolar disorder | 296.00–05, 296.10, 296.14, 296.40–45, 296.50–55, 296.60–64, 296.7, 296.80–82, 296.89 |
| Delirium/dementia | 293.0–1, 294.0, 294.8–9, 301.0, 310.2, 310.89, 310.9 |
| Depression | 293.83, 296.20–26, 296.30–36, 300.4, 311 |
| Drug abuse/dependence | 304.00–03, 304.10–13, 304.20–23, 304.30–33, 304.40–43, 304.50, 304.53, 304.60–63, 304.70, 304.71–73, 304.80–83, 304.90–93, 305.20–23, 305.30, 305.32–33, 305.40–43, 305.50–53, 305.60–63, 305.70–73, 305.80–81, 305.90–93 |
| Personality disorders | 301.0, 301.10, 301.12–13, 301.20, 301.22, 301.3–4, 301.50–51, 301.59, 301.6–7, 301.81–83, 301.89, 301.9 |
| PTSD | 309.81 |
| | |
| Exploratory LLB-related Diagnoses | |
| Post-concussive syndrome | 310.2 |
| Tinnitus | 338.3 |
| Fatigue | 780.7 |
| Migraines | 346 |
